# A Bayesian spatio-temporal model of COVID-19 spread in England

**DOI:** 10.1038/s41598-024-60964-0

**Published:** 2024-05-06

**Authors:** Xueqing Yin, John M. Aiken, Richard Harris, Jonathan L. Bamber

**Affiliations:** 1 School of Geographical Sciences, University of Bristol, Bristol, BS8 1SS UK; 2 Expert Analytics, 0179 Oslo, Norway; 3 Njord Centre, Departments of Physics and Geosciences, University of Oslo, 0371 Oslo, Norway; 4 Department of Aerospace and Geodesy, Technical University of Munich, 80333 Munich, Germany

**Keywords:** Risk factors, Epidemiology, Statistics

## Abstract

Exploring the spatio-temporal variations of COVID-19 transmission and its potential determinants could provide a deeper understanding of the dynamics of disease spread. This study aimed to investigate the spatio-temporal spread of COVID-19 infections in England, and examine its associations with socioeconomic, demographic and environmental risk factors. We obtained weekly reported COVID-19 cases from 7 March 2020 to 26 March 2022 at Middle Layer Super Output Area (MSOA) level in mainland England from publicly available datasets. With these data, we conducted an ecological study to predict the COVID-19 infection risk and identify its associations with socioeconomic, demographic and environmental risk factors using a Bayesian hierarchical spatio-temporal model. The Bayesian model outperformed the ordinary least squares model and geographically weighted regression model in terms of prediction accuracy. The spread of COVID-19 infections over space and time was heterogeneous. Hotspots of infection risk exhibited inconsistent clustering patterns over time. Risk factors found to be positively associated with COVID-19 infection risk were: annual household income [relative risk (RR) = 1.0008, 95% Credible Interval (CI) 1.0005–1.0012], unemployment rate [RR = 1.0027, 95% CI 1.0024–1.0030], population density on the log scale [RR = 1.0146, 95% CI 1.0129–1.0164], percentage of Caribbean population [RR = 1.0022, 95% CI 1.0009–1.0036], percentage of adults aged 45–64 years old [RR = 1.0031, 95% CI 1.0024–1.0039], and particulate matter ($\text {PM}_{2.5}$) concentrations [RR = 1.0126, 95% CI 1.0083–1.0167]. The study highlights the importance of considering socioeconomic, demographic, and environmental factors in analysing the spatio-temporal variations of COVID-19 infections in England. The findings could assist policymakers in developing tailored public health interventions at a localised level.

## Introduction

Coronavirus Disease 2019 (COVID-19) has caused an enormous challenge to global public health, social security and the economy. The disease originated in Wuhan in China in December 2019, and was declared a pandemic by the World Health Organisation (WHO) in March 2020^1^. As of February 2023, over 650 million people have been infected by the virus across the world, and the global death toll linked to COVID-19 has been recorded at over 6.7 million (https://covid19.who.int/). Although vaccination and public health measures have been successful in reducing the spread of COVID-19 in many areas, gaining a comprehensive understanding of the spatio-temporal transmission patterns and key determinants of the virus remains crucial in the ongoing public health fight against this global health threat. Recent research studies have proposed that COVID-19 transmission and severity may be influenced by various risk factors. Some researchers examined the association of COVID-19 mortality and infections data with socioeconomic status (e.g., deprivation index, income and unemployment), and found that the virus hit harder in socioeconomically disadvantaged communities^2–6^. Some researchers indicated that environmental conditions such as air pollution, humidity and temperature could influence the virus transmission^7–11^. In addition, there have been papers on investigating the demographic influences (e.g., race, gender, age distribution) of COVID-19 transmission^12–20^. However the available evidence on these determinants to date presents a mixed and inconsistent picture. For example, Castr et al.^21^ found a strong positive relationship of COVID-19 incidence and mortality with income, whereas Liu et al.^22^ showed that higher income groups demonstrated lower incidence rates. A multi-country study carried out by Huang et al.^23^ found significant effects of $\text {PM}_{2.5}$ on COVID-19 incidence in the USA but not in Italy or Canada. Furthermore, few studies have accounted for socioeconomic, demographic and environmental factors simultaneously to explain the spatio-temporal variations of COVID-19 infection. The simultaneous consideration of these factors is important for several reasons. Firstly, it provides a more holistic understanding of the pandemic’s complexities between these variables and how they contribute to the spatio-temporal variation in COVID-19. Secondly, previous studies^2,8,9,16,18^ have shown that COVID-19 transmission is influenced by multiple factors, therefore by considering these factors together, researchers can better control for potential confounding variables, which leads to more accurate and robust results. Thirdly, considering socioeconomic, demographic and environmental factors simultaneously allows policymakers to implement targeted interventions and effective response strategies that address specific vulnerabilities and mitigate the impact of the virus more effectively compared to approaches that only consider one factor. Therefore, this paper adds to the global evidence base on the topic of identifying influencing factors of COVID-19 spread, by presenting a study that investigates the spatio-temporal spread of COVID-19 infection risk and its associations with socioeconomic, demographic and environmental factors in England. Our study is based on aggregated count data summarising COVID-19 infected cases between 7 March 2020 and 26 March 2022 at Middle Layer Super Output Area (MSOA) scale in England and by week. The data are modelled using a Bayesian hierarchical model, where the spatio-temporal variation in COVID-19 transmission is explained by a set of risk factors of interest, important confounding factors, and random effects that account for the spatio-temporal autocorrelation in the data.

To the best of our knowledge, this study represents the first attempt to incorporate socioeconomic, demographic and environmental factors simultaneously to analyse the spread of COVID-19 infections at MSOA level across England. Furthermore, it is one of the most up-to-date and comprehensive studies in terms of its temporal duration. For example, Sun et al.^2^ used data up to May 2020, Sartorius et al.^24^ used data up to 23 August 2020, while the data used by Harris & Brunsdon^16^ goes up to 21 May 2021. In addition, compared to previous studies that mostly used frequentist approaches, such as the geographically weighted regression^21,25,26^, spatial error model^18,27,28^ and spatial lag model^29–31^, to identify the drivers of the spread of COVID-19, the Bayesian hierarchical model developed here has several advantages. Firstly, it allows for the incorporation of prior knowledge by specifying prior distributions for the model parameters through a hierarchical modelling scheme^32–34^. Secondly, rather than providing a single estimate for the regression coefficients in the model, Bayesian regression provides a posterior distribution of the parameters, which allows for evaluation of its uncertainty and significance, as well as a more robust assessment of the uncertainties in risk estimates. Thirdly, the model accounts for spatio-temporal autocorrelation in the data by modelling the random effects via a continuous Gaussian random field process. Thus it is able to account for missing case data by borrowing information from nearby locations in space and/or time based on the modelled spatio-temporal dependence structure. This enables predictions of COVID-19 infection rates in any geographical location and at any time point, even if the infection cases have not been recorded. This is important from a public health perspective, because COVID-19 is often asymptomatic globally, especially during the early phase of the pandemic when there was a lack of resources to facilitate large-scale testing^16,35^. Therefore, the ability of the model to predict the COVID-19 risk even in areas where data are incomplete could provide decision-makers with more comprehensive and accurate information to guide interventions and resource allocation in the fight against the pandemic. This study quantifies the spatio-temporal patterns and disparities in COVID-19 infections across England, while also identifying hotspot areas and providing additional evidence on the influencing factors of COVID-19 spread. The Bayesian model utilised in this analysis has demonstrated its effectiveness in accurately predicting COVID-19 infection risk, by exhibiting better modelling accuracy than the ordinary least squares (OLS) and geographically weighted regression (GWR) models. The obtained findings provide valuable insights for policymakers to optimise healthcare resources, establish targeted public health interventions, and improve epidemic prevention and control systems.

## Methods

### Study region

**Figure 1:**
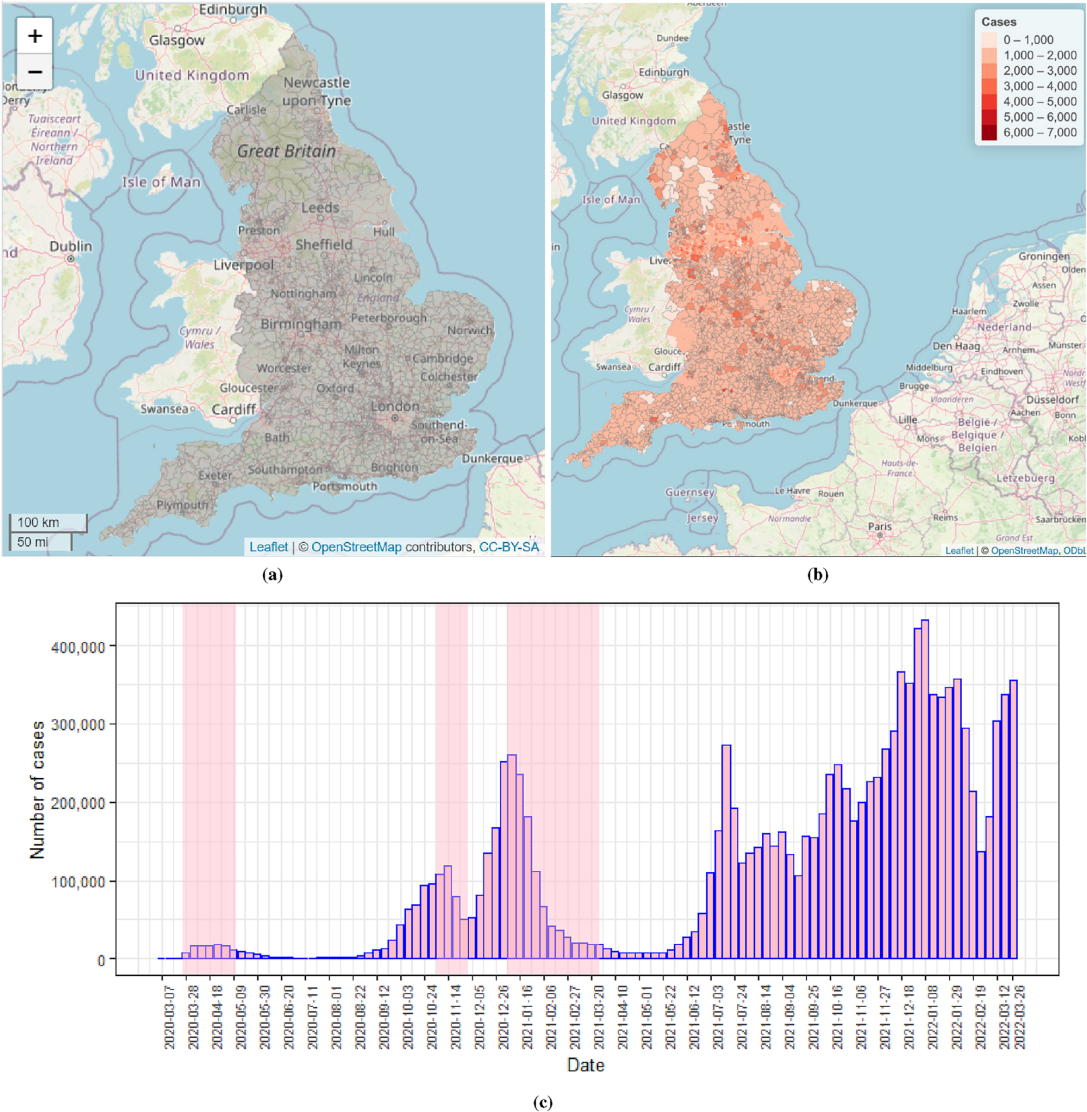
Maps of the study region (**a**) and cumulative number of COVID-19 infection cases (**b**) at English MSOA level during the study period. (**c**): Plot of the reported number of COVID-19 cases by week for all MSOAs in mainland England between 7 March, 2020 and 26 March, 2022. Weeks of lockdown are highlighted in pink. Note that some MSOAs, such as these around Liverpool, Sheffield and London, have a highly concentrated number of MSOA boundaries. In order to enhance the overall readability of the visualization and avoid an overly cluttered map, we simplified the representation in these highly congested areas by selectively removing some of the MSOA boundaries that were closely clustered together in Figure 1 (b)..

The study region, as shown in Fig. 1a, is mainland England, which is partitioned into $n= 6789$ small neighbourhood units called Middle Layer Super Output Areas (MSOAs). MSOAs are a statistical geography which are designed by English Government for reporting small-area statistics. They represent a formal, administrative specification of neighbourhoods and are developed with specific criteria, including broadly equal population size, socio-economic similarity based on accommodation type and tenure, and spatial compactness of the zones^36^. The shapefiles for MSOAs were obtained from the open geoportal platform (https://geoportal.statistics.gov.uk/) provided by the UK government. The median geographical size of an MSOA in England is 3.04 $\text {km}^2$, and as of the end of June 2020, the population size in a single MSOA ranges between 4,843 and 27,911 persons, with a median population size of 8,123.

### COVID-19 data

The COVID-19 infection data used for this study were obtained from the official UK Government COVID-19 dashboard (https://coronavirus.data.gov.uk/details/download) on the weekly number of reported COVID-19 cases for each MSOA. The infections data tally, by date reported, the number of individuals who had at least one positive COVID-19 test result, over that and the preceding six days. The observed time frame of the study spanned $T=108$ weeks from the seven days leading up to 7 March 2020 (week 1) to the seven days to 26 March 2022 (week 108). The data only include pillar 1 cases until 2 July 2020, from when pillar 2 cases are also included. Pillar 1 cases come from the swab testing carried out in Public Health England lab and NHS hospitals for those with a clinical need, and health and care workers, while pillar 2 cases are also swab tests but done for the wider population. These tests are done at local and regional test sites, mobile testing units, satellite test centres and via home tests (https://www.gov.uk/government/publications/nhs-test-and-trace-statistics-england-methodology/nhs-test-and-trace-statistics-england-methodologyhttps://www.gov.uk/government/publications/nhs-test-and-trace-statistics-england-methodology/). A total of 12,097,525 cases (MSOA and week combinations) were reported during the entire study period, whose values ranged from 3 to 978 cases in a single MSOA and week with a median of 28 cases. Note that the positive cases per MSOA range from 3 upwards is because of censoring of smaller numbers (between 0 and 2 cases) in the reported data. Figure 1b displays the spatial pattern of the cumulative COVID-19 infection cases over all weeks at English MSOA level, with higher numbers of infected cases in and around major metropolitan areas such as Newcastle, Manchester, Liverpool, Birmingham, Leicester, Sheffield, and London. Figure 1c presents the weekly number of reported COVID-19 cases for all MSOAs, with the x-axis labeled “date” indicating the start date of each observation week. During the COVID-19 pandemic, the UK government introduced various measures, such as the tiered system, the localise and nationwide lockdowns to limit the spread of COVID-19. Our observation window encompasses three separate periods of national lockdown in England, which were officially announced and took place from 26 March to 12 May 2020, 5 November to 2 December 2020, and 5 January to 28 March 2021, respectively^37^. To aid in visualising these periods, they have been shaded in pink in the figure. The figure shows that the first wave of the COVID-19 outbreak in England started in March 2020 and ended at the end of May 2020, whereas the second wave started at the beginning of September 2020 and then reached an initial peak in mid-November. After that the infection levels declined before drastically rising again in late December of 2020 and peaking in early January 2021. There were signs of decrease in the week starting 9 January 2021 until early June, after which the infections started to rise and then wavered until the end of March 2022, with the highest numbers of confirmed cases (> 400,000 cases) observed in early January 2022 (i.e., the week beginning 8 January 2022). Overall, there was typically a reduction in the number of infection cases after the national lockdowns were in place, particularly during the second and third lockdown periods.

### Socioeconomic, demographic and environmental variables

 A variety of socioeconomic, demographic and environmental factors were selected as potential explanatory variables for the spread of COVID-19 infections, and all these variables were retrieved or prepared at MSOA level. Based on existing studies and data availability, our socioeconomic variables include annual household income, which was available for 2018 (https://www.ons.gov.uk/employmentandlabourmarket/peopleinwork/earningsandworkinghours), and unemployment rate, which was measured as the percent of people aged 16 years and over unemployed in the labour force, and was available from the 2021 UK census (https://www.nomisweb.co.uk/census/2021/bulk) held by the Office for National Statistics (ONS). The demographic factors of population density, ethnic and age distributions by MSOA were also available from the 2021 UK census. Population density was measured as the number of persons per square kilometer, and it was found to be highly skewed to the right. Thus a log-transformation was applied. Environmental variables include the annual mean concentrations of fine particulate matter ($\text {PM}_{2.5}$), coarse particulate matter ($\text {PM}_{10}$) and nitrogen dioxide ($\text {NO}_{2}$) for 2021, all of which were measured in µg $\text {m}^{-3}$. We used concentrations data for 2021 rather than for 2022 or 2020, because the data for 2022 were not available, while the data for 2020 were artificially lower than normal concentration levels due to the implementation of lockdowns and mobility restrictions across England in 2020, which significantly reduced transport usage and hence pollution concentrations of that year. Department for the Environment, Food and Rural Affairs (DEFRA) offers 1 $\times$ 1 km gridded annual mean $\text {PM}_{2.5}$, $\text {PM}_{10}$ and $\text {NO}_{2}$ concentrations (https://uk-air.defra.gov.uk/data/pcm-data), which is spatially misaligned with the irregularly shaped MSOAs. Thus we adopted the simple averaging method^38^ to obtain the concentrations by MSOA. Specifically, each 1 $\text {km}^2$ gridded concentration has an associated centroid, and the pollution concentration in an MSOA is computed by averaging the grid square concentrations whose centroids are located within the MSOA. If an MSOA does not contain a grid square centroid, it is assigned the pollution concentration from the nearest grid square. Table 1 lists the variables together with their source of data. Finally, we also considered two other confounding factors that may impact COVID-19 ill health, including the number of care home beds per adult population (https://www.cqc.org.uk/), and a binary variable indicating whether there is a hospital with emergency facilities or not in an MSOA (https://www.nhs.uk/).

**Table 1:** Summary of the socioeconomic, demographic and environmental variables used in this study.

Theme	Variable	Year	Source
Socioeconomic	Annual household income (in thousand, $\pounds$)	2018	ONS
	Unemployment rate (%)	2021	ONS
Demographic	Log(Population density)	2021	ONS
	Percent of Chinese (%)	2021	ONS
	Percent of Indian (%)	2021	ONS
	Percent of Pakistani (%)	2021	ONS
	Percent of Bangladeshis (%)	2021	ONS
	Percent of African (%)	2021	ONS
	Percent of Caribbean (%)	2021	ONS
	Percent of white British (%)	2021	ONS
	Percent of age 18–29 (%)	2021	ONS
	Percent of age 30–44 (%)	2021	ONS
	Percent of age 45–64 (%)	2021	ONS
	Percent of 65 years old and over (%)	2021	ONS
Environmental	Annual mean $\text {PM}_{2.5}$ (µg $\text {m}^{-3}$)	2021	DEFRA
	Annual mean $\text {PM}_{10}$ (µg $\text {m}^{-3}$)	2021	DEFRA
	Annual mean $\text {NO}_2$ (µg $\text {m}^{-3}$)	2021	DEFRA

### Spatial and temporal autocorrelation analysis

 Naively using the raw number of confirmed COVID-19 cases or the proportion of the positive cases in a population as a measure of the variations in COVID-19 transmission ignores the spatio-temporal autocorrelation that characterises the disease dynamics. Moreover, it also ignores the potential effects of risk factors on COVID-19 infections. Therefore, it is necessary to develop a model-based approach that can capture the spatio-temporal variations of the disease spread, separate the variations from random noise and account for the spatio-temporal autocorrelation structure in the data. The presence of positive spatial autocorrelation in the COVID-19 infections data was evidenced by performing the Moran’s I test^39^, which is widely used to measure the level of spatial autocorrelation between adjacent locations globally. The computed Moran’s I value was 0.242 and the associated *P* value was less than 0.0001, indicating statistically significant spatial correlation in the COVID-19 spread throughout England. To assess the temporal autocorrelation in the data, a Ljung-Box test^40^ was conducted. The test showed strong evidence of positive temporal correlation in the COVID-19 cases over time, as indicated by a *P* value of less than 0.0001.

### Variable selection

 Before modelling the data, we examined the multicollinearity among the variables described in Table 1 as well as the confounding risk factors using a Pearson’s correlation analysis, because the presence of multicollinearity among independent variables can cause overfitting and less reliable inferences about the associations between the response and predictor variables. The air quality components $\text {PM}_{10}$ and $\text {NO}_{2}$ were found to be strongly correlated with $\text {PM}_{2.5}$, with the Pearson’s correlation coefficients of 0.86 and 0.85, respectively. Given that previous studies have identified $\text {PM}_{2.5}$ as an important risk factor for COVID-19 infections^9,11,41^, we selected $\text {PM}_{2.5}$ over the other two air pollutants for further analysis. The percent of each MSOA’s population who were white British was highly correlated with the percent of African and Indian population, with the correlation coefficients of − 0.7 and − 0.6 respectively, and hence was not included in the analysis. The percent of the population between 30 and 44 years old was also not considered in the model due to its high correlations with the percent of age groups 45–64, and 65 years and over, with the correlation coefficients of − 0.6 and − 0.8, respectively. Simultaneously, the variance inflation factor (VIF) was used to verify multicollinearity. The VIF values for all the filtered variables were less than 5 (between 1.02 and 4.89), indicating no serious multicollinearity exists^42^. This means that all the predictor variables in the final model were not highly correlated to each other.

### Bayesian spatio-temporal modelling

 We employed a Bayesian hierarchical model to predict the COVID-19 infection risk at MSOA level over space and time and identify the associated risk drivers, by utilising a generalised linear mixed model with a combination of the selected risk factors, and random effects which account for any residual spatio-temporal dependence of COVID-19 transmission. In this study, we define the COVID-19 infection risk (or rate) as the ratio of the number of reported COVID-19 cases to the total population. Let $Y({\varvec{s}}_{i},t)$ and $N({\varvec{s}}_{i},t)$ be the number of reported COVID-19 cases and total population in MSOA $i\in (1, \ldots , n_t)$ during week $t \in (1,\ldots ,T)$, respectively. Here ${\varvec{s}}_{i} \in R^2$ denotes the geographical location for MSOA *i*, and $n_t$ represents the number of MSOAs that have reported COVID-19 cases during week *t*. As the outcome variable $Y({\varvec{s}}_{i},t)$ is a count, the data likelihood model takes Poisson distributions. The first level of the Bayesian hierarchical model is the Poisson log-linear specification given by

1 $$\begin{aligned} Y({\varvec{s}}_{i},t)&\sim \text {Poisson}(\mu ({\varvec{s}}_{i},t)),\ \ i=1,\ldots ,n_t;\ \ t=1,\ldots ,T,\nonumber \\ \mu ({\varvec{s}}_{i},t)&= N({\varvec{s}}_{i},t)\times \theta ({\varvec{s}}_{i},t),\nonumber \\ \ln (\theta ({\varvec{s}}_{i},t) )&=\varvec{x}({\varvec{s}}_{i})^{\top } \varvec{\beta }+\xi ({\varvec{s}}_{i},t). \end{aligned}$$

Here $\mu ({\varvec{s}}_{i},t)$ is the Poisson mean, which is a product of $N({\varvec{s}}_{i},t)$, the total number of populations in MSOA *i* during week *t*, and $\theta ({\varvec{s}}_{i},t)$, the underlying unknown COVID-19 infection risk in MSOA *i* and week *t*. The log-infection risk is modelled by two components, the first of which is the vector of *p* known covariates $\varvec{x}({\varvec{s}}_{i})=(1, x_1({\varvec{s}}_{i}),\ldots , x_p({\varvec{s}}_{i}))$ related to location ${\varvec{s}}_{i}$, including an intercept term, with regression parameters $\varvec{\beta }=(\beta _0,\beta _1,\ldots ,\beta _p)$. As the temporally varying covariate information is not available, we cannot include this in the model and the regression parameters are assumed unchanged over time. The second component accounting for the variations in $\mu ({\varvec{s}}_{i},t)$ is the spatio-temporal random effect $\xi ({\varvec{s}}_{i},t)$, which represents the realization of a spatio-temporal process for the logarithm of COVID-19 infection risk after covariate adjustment. The spatial correlation is modelled by location specific random effects through a Gaussian random field process, which captures the correlation via a covariance matrix expressed as a function of distance between locations. The temporal correlation is modelled by a first-order autoregressive (AR1) process. Specifically, the second level of the model is given by

2 $$\begin{aligned} \xi ({\varvec{s}}_{j},t)&=\alpha \times \xi ({\varvec{s}}_{j},t-1)+\omega ({\varvec{s}}_{j},t), \text { and } \xi ({\varvec{s}}_{j},1)\sim \text {N}\left( 0,\frac{\sigma _{\omega }^2}{1-\alpha ^2}\right) , \end{aligned}$$

where $\alpha$ is a temporal dependence parameter such that $\vert \alpha \vert < 1$. The temporal dependence parameter quantifies the strength and direction of the relationship between the current and past values of the time series. Specifically, in this study $\alpha$ represents the influence of the value $\xi ({\varvec{s}}_{j}, t-1)$ at time $t-1$ on the subsequent value $\xi ({\varvec{s}}_{j}, t)$ at time *t*. Values of $\alpha$ close to 1 indicate strong positive temporal dependence, values of $\alpha$ near 0 suggest little or no temporal dependence, and values close to − 1 indicate strong negative temporal dependence. $\varvec{\omega }({\varvec{s}}_{j},t)=\left( \omega ({\varvec{s}}_{1},t), \ldots , \omega ({\varvec{s}}_{n_t},t)\right) ^{\top }$ is a spatial random effect that is assumed to follow a multivariate Gaussian distribution and have $\varvec{\omega }({\varvec{s}}_{j},t) \sim \text {N}(\varvec{0}_{n_t}, \sigma _{\omega }^2\varvec{\Sigma }_{\omega })$, where $\varvec{0}_{n_t}$ is a $n_t \times 1$ vector of zeros and $\sigma _{\omega }^2$ is the marginal variance of the spatial process. $\varvec{\Sigma }_{\omega }$ is the $n_t \times n_t$ covariance matrix with elements $(\varvec{\Sigma }_{\omega })_{ij}=C(\vert \vert \varvec{s}_{i}-\varvec{s}_{j}\vert \vert ),$ where $\vert \vert \varvec{s}_{i}-\varvec{s}_{j}\vert \vert$ is the distance between locations $(\varvec{s}_i, \varvec{s}_j)$, and $C(\cdot )$ is the Matern function^43^ given by

3 $$\begin{aligned} C(\vert \vert \varvec{s}_{i}-\varvec{s}_{j}\vert \vert )= \frac{1}{2^{\nu -1}\Gamma (\nu )}(\kappa ||\varvec{s}_i-\varvec{s}_j||)^{\nu }K_{\nu }(\kappa ||\varvec{s}_i-\varvec{s}_j||), \end{aligned}$$

where $K_{\nu }(\cdot )$ is the modified Bessel function of second kind, and $\Gamma (\nu )$ is the Gamma function. $\nu$ is the smoothness parameter of the Matern covariance function, which is fixed at 1. $\kappa$ is a scaling parameter controlling the spatial correlation range $\rho$, which is the distance at which the correlation function has fallen to about 0.1 and is given by $\rho =\sqrt{8\nu }/\kappa$.

The third level of the model specifies the prior specifications for the model parameters. The regression parameters $\varvec{\beta }=(\beta _0, \beta _1, \ldots , \beta _p)$ were assigned independent weakly informative zero-mean Gaussian prior distributions with a large variance, i.e., $\beta _j \sim \text {N}(0, 1000)$, to ensure their values are mainly informed by data. Penalised complexity priors^44^ were specified for the correlation range $\rho$ and the marginal standard deviation parameter $\sigma _{\omega }$, with $p(\rho <1.5)= 0.5$ and $p(\sigma _{\omega }>1)=0.01$, indicating a 0.5 probability of $\rho$ being smaller than 1.5, and a low probability of $\sigma _{\omega }$ being greater than 1, respectively. The temporal autoregressive parameter $\alpha$ was also assigned a penalised complexity prior, with $p(\alpha >0)=0.9$. Finally, a weakly informative log-gamma prior was specified for $\sigma _{e}^2$, i.e., $\ln (\sigma _{e}^2) \sim \text {log-Gamma}(1, 0.00005)$.

All analyses were conducted using the statistical software R version 4.2.1^45^. Model inference was implemented using the stochastic partial differential equations (SPDE) method and the Integrated Nested Laplace Approximations (INLA) algorithm via the R-inlabru package (2.5.2)^46–48^. This approach has significant advantages in terms of computational efficiency and accuracy when handling high-resolution spatio-temporal processes and large datasets. The INLA implementation of the SPDE approach approximates a continuous Gaussian random field process with the Matern covariance function by a discretely indexed spatial random process known as a Gaussian Markov random field (GMRF). The GMRF has zero mean and uses a sparse precision matrix, which thus substantially reduces the computational cost in matrix algebra operations compared to using dense covariance matrices^49^. To represent the Matern field as a GMRF, the SPDE approach discretizes the space by defining a finite element mesh composed of non-intersecting triangles that partition the domain of the study area^47^. These triangles allow the spatial autocorrelation between observations to be calculated in the modelling process. Then the INLA algorithm estimates the posterior distribution of the latent Gaussian process and hyperparameters using the Laplace approximation^46^.

Other R packages including spdep (1.2.5)^50^, ggplot2 (3.3.6)^51^, rgdal (1.5.32)^52^, dplyr (1.0.10)^53^, RColorBrewer (1.1.3)^54^, leaflet (2.1.1)^55^, and stats (4.2.1) have also been used for data analysis and visualisation. Model parameter estimates were summarised in posterior mean, standard deviation (SD) and 95% credible intervals. A risk factor is considered to have a statistically significant effect if the 95% credible interval of its estimated regression coefficient does not include zero. The main code to complete this analysis is available from https://github.com/XueqingYin/COVID-19.git. In addition, for comparison purpose, the OLS and GWR models were also fitted to the same data to observe which one produces the most accurate predictions of infection risk, i.e., $\theta ({\varvec{s}}_{i},t)$.

Ordinary least squares (OLS). The OLS model is a regression model that investigates the relationships between a dependent variable and a set of explanatory variables. It has the general form of

$$\begin{aligned} \ln \left( \frac{Y({\varvec{s}}_{i},t)}{N({\varvec{s}}_{i},t)}\right) =\varvec{x}({\varvec{s}}_{i})^{\top }\varvec{\beta }+\epsilon _{it}, \ \ i=1,\ldots ,n_t;\ \ t=1,\ldots ,T, \end{aligned}$$

where $\frac{Y({\varvec{s}}_{i},t)}{N({\varvec{s}}_{i},t)}$ is the observed COVID-19 infection risk in MSOA *i* and week *t*. OLS optimises the regression coefficients $\varvec{\beta }=(\beta _0,\beta _1,\ldots ,\beta _p)$ by minimizing the sum of squared prediction errors^56^. It assumes that the observations are independent across the study area and time, and the error terms are not correlated^57^. Therefore OLS assumes that the observations at MSOA level are independent of each other and does not consider any spatial and temporal dependence.

### Geographically weighted regression (GWR)

 OLS assumes the relationships between explanatory variables and dependent variables do not vary over space. In contrast, GWR allows these relationships to vary over space, and the regression parameters to be derived for each location separately, which incorporates the geographic context^58^. It has the general form of

$$\begin{aligned} \ln \left( \frac{Y({\varvec{s}}_{i},t)}{N({\varvec{s}}_{i},t)}\right) =\varvec{x}({\varvec{s}}_{i})^{\top }\varvec{\beta }({\varvec{s}}_{i})+\epsilon _{it}, \ \ i=1,\ldots ,n_t;\ \ t=1,\ldots ,T, \end{aligned}$$

where $\varvec{\beta }({\varvec{s}}_{i}) =(\beta _0({\varvec{s}}_{i}), \beta _1({\varvec{s}}_{i}),\ldots , \beta _p({\varvec{s}}_{i}))$ is related to location $\varvec{s}_{i}$, including an intercept term $\beta _0({\varvec{s}}_{i})$.

### Measuring the prediction performance

To quantify the prediction accuracy of each model, statistical measures including root mean square error (RMSE) and mean absolute error (MAE) for the infection risk predictions were computed. Their mathematical equations are as follows:

4 $$\begin{aligned} \text {RMSE}&=\sqrt{\frac{1}{n_{\text {obs}}}\sum _{t=1}^{T}\sum _{i=1}^{n_t}(\hat{\theta }({\varvec{s}}_{i},t) -\theta ({\varvec{s}}_{i},t) )^2}, \end{aligned}$$

5 $$\begin{aligned} \text {MAE}&=\frac{1}{n_{\text {obs}}}\sum _{t=1}^{T}\sum _{i=1}^{n_t}\vert \hat{\theta }({\varvec{s}}_{i},t)-\theta ({\varvec{s}}_{i},t)\vert , \end{aligned}$$

where $\theta ({\varvec{s}}_{i},t)$ and $\hat{\theta }({\varvec{s}}_{i},t)$ represent the observed and predicted values at a given location ${\varvec{s}}_{i}$ and time *t*, and $n_{\text {obs}}$ represents the total number of observations across all time steps. A model with lower RMSE and MAE values has higher prediction accuracy.

### K-means clustering

 Clustering methods can be used to identify the groups of MSOAs with high and low infections, and in this study k-means clustering was utilised. K-means clustering^59^ assigns each object (MSOA) to its closet centroid (center) by the Euclidean distance, and the collection of objects assigned to the same centroid forms a cluster. A centroid is defined as the average of all the objects in the cluster. The goal of this method is to minimize the within-cluster sum-of-squares. Suppose all MSOAs are partitioned into *K* clusters. The basic k-means clustering algorithm for generating a cluster structure with *K* clusters is outlined as follows.

**Algorithm 1:**
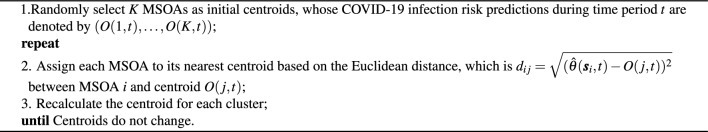
Basic k-means clustering algorithm.

### Computing COVID-19 infection risk by region

 The 6789 MSOAs are nested exactly within nine regions of England, thus in this study, we also analyse the evolution of infection risk over time in each region. We computed the weighted averages of estimated infection risks for region *r* and week *t* as

6 $$\begin{aligned} \hat{\phi }_{rt}= \frac{\sum _{i\in r} p_{i}\hat{\theta }({\varvec{s}}_{i},t)}{p_r}, \end{aligned}$$

where $\hat{\theta }({\varvec{s}}_{i},t)$ denotes the estimated infection risk in MSOA *i* and week *t*, $p_{i}$ and $p_{r}$ indicate the population sizes of MSOA *i* and region *r* respectively, and $i \in r$ means that MSOA *i* is geographically located within region *r*. We used population size as the weighting factor in the computation due to the assumption that neighbourhoods with larger populations are likely to make a greater contribution to the overall infection level of a region compared to those with smaller populations.

## Results

### Prediction performance

The proposed model was applied to the COVID-19 infections data. For comparison purpose, the OLS and GWR models were also fitted to the same dataset to observe which one produces the most accurate predictions of infection risk, i.e., $\theta ({\varvec{s}}_{i},t)$. The OLS model is the most common and widely used global non-spatial regression model, while the GWR model is a spatial model that allows the relationships between the independent and dependent variables to vary by locality. Given the spatial nature of the GWR model, it was applied separately to the COVID-19 infections data for each time period, without considering the temporal autocorrelation in the data. To quantify the prediction accuracy of each model, statistical measures including root mean square error (RMSE) and mean absolute error (MAE) for the infection risk predictions were computed.

It can be seen from Table 2 that our spatio-temporal Bayesian model (denoted ST-Bayesian) performed best in terms of prediction accuracy. The RMSE value for the Bayesian model was 72.5% lower than that of the OLS model and 15.4% lower than that of the GWR model. The MAE value for the Bayesian model outperformed the OLS model by 74.1% and the GWR model by 12.5%. To ensure a fair comparison with the GWR model, we additionally applied a purely spatial version of the ST-Bayesian model (denoted S-Bayesian) to the COVID-19 infections data for each time period without considering the temporal autocorrelation. The S-Bayesian model also produced more accurate risk predictions than the GWR model due to its lower RMSE values. Although the ST-Bayesian and S-Bayesian model exhibited similar performance, the ST-Bayesian model is more appropriate in this research context. This is because the ST-Bayesian model is capable of quantifying the temporal dependence of the disease transmission over time, which is not feasible in a purely spatial model. This temporal aspect is crucial for understanding the dynamics of COVID-19 and predicting its future spread, and is also a key benefit of the approach we demonstrated.

**Table 2:** Prediction accuracy of different models for the COVID-19 risk.

Model	RMSE	MAE
ST-Bayesian	0.0011	0.0007
OLS	0.0040	0.0027
GWR	0.0013	0.0008
S-Bayesian	0.0011	0.0008

Therefore, the subsequent sections will present the primary results obtained from the proposed spatio-temporal Bayesian model, because it is the best fitting model in terms of model accuracy. To explore the transmission of COVID-19 at MSOA level and understand its relationships with socioeconomic, demographic and environmental risk factors, we will focus on the following research questions of interest.

1. Were there health inequalities in COVID-19 infections among MSOAs in mainland England, and how did these inequalities evolve over time?
2. What impacts did the socioeconomic, demographic and environmental factors have on COVID-19 risk?
3. Where were the hotspots of COVID-19?
4. How did the COVID-19 risk change over time in different regions of England?

### Overall health inequalities and temporal evolution of COVID-19 risk

Table 3 provides a summary of the estimates for the spatial correlation range parameter $\rho$ and temporal dependence parameter $\alpha$ from the model. It indicates that the spread of COVID-19 infections in England had a positive spatial and temporal autocorrelation, with $\rho =63.048$ (SD = 0.444, 95% CI 62.049–63.825) and $\alpha =0.908$ (SD = 0.003, 95% CI 0.905–0.913). The spatial dependence remains significant for up to 63 kilometers. These estimates lend further support to the choice of a spatio-temporal model as an appropriate framework for modelling the spread of COVID-19 infections.

**Table 3:** Summary of the spatial correlation range parameter $\rho$ and the temporal dependence parameter $\alpha$.

Parameter	Mean	SD	0.025quant	0.975quant
Spatial range $\rho$ (kilometers)	63.048	0.444	62.049	63.825
Temporal dependence $\alpha$	0.908	0.003	0.905	0.913

**Figure 2:**
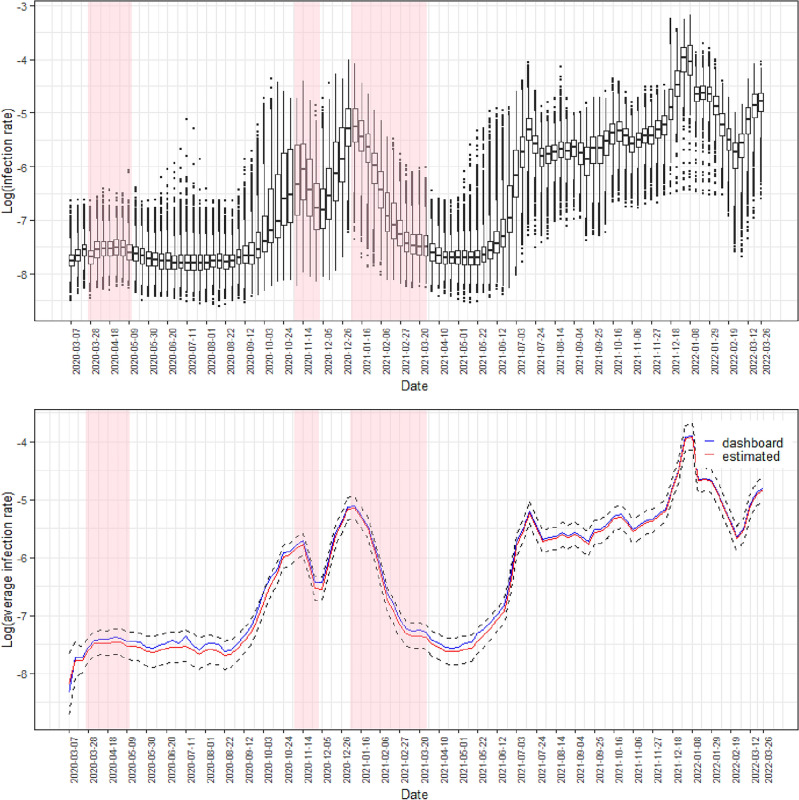
Summary of the estimated COVID-19 infection risk. The top panel displays boxplots of the logarithm of the estimated infection rates across all MSOAs over the 108 weeks, while the bottom panel compares the model’s average estimated rate on the log scale across mainland England (with 95% credible intervals represented by dashed lines) to the log of the average rate computed based on UK COVID-19 dashboard reported cases in weeks. The x-axis label “date” indicates the starting date of each observation week. Weeks of lockdown are highlighted in pink.

Figure 2 shows two panels illustrating the estimated COVID-19 risk for all MSOAs by week. To improve the data visibility, we chose to plot the logarithm of the risk. The top panel reveals the presence of health inequalities in COVID-19 risk in England. There were substantial variations in the estimated risks and the extent of these spatial inequalities increased over time, because the interquartile range of the risk, which measures the spread of the distribution, widened from 0.0001 in the first observation week (starting on 7 March 2020) to 0.0028 in the last observation week (starting on 26 March 2022), indicating a potentially growing disparity in COVID-19 infections across different neighbourhoods over time.

The bottom panel in Fig. 2 shows that the average COVID-19 risk estimated by our model were highly consistent with the UK COVID-19 dashboard reported data. Over the study period, the COVID-19 infection risk displayed a series of fluctuations, but there was an overall upward temporal trend. The estimated COVID-19 rates ranged from 0.0002 to 0.0417, with a mean of 0.0028 (SD = 0.0037). The average infection risk curve shows an initial peak in mid-November 2020, followed by a decline in infection levels, which then surged in December 2020 due to the Alpha (also known as B.1.1.7) variant of coronavirus. Infections peaked in early January 2021, and then tapered off to almost baseline levels by April of the same year. From the week of 5 June 2021 to mid-July, the risk began to rise again, reaching an estimated 0.5%. The rate then fell and displayed a relatively stable trend with slight oscillations until November 2021. However, the risk reached its highest point in the week of 1 January 2022 at 1.9%, before substantially decreasing until early March 2022. The curve also suggests that the national lockdowns reduced the COVID-19 risk, particularly during the second and third lockdown periods, while individuals were at a higher risk of COVID infection during the months of July, December, and January. We also noted that in the first weeks of the study period both the estimated risks and their variations across MSOAs were quite low. This is likely because during the first wave of the pandemic between March and July 2020, COVID-19 testing capacity was strictly limited to priority groups due to a lack of infrastructure for large-scale testing, which means that a large number of infected individuals were not formally diagnosed with the virus. As a result, data on confirmed COVID-19 cases were incomplete, thus leading to underestimated infection risk and small variation. We observed that our model tends to slightly underestimate the infection risk. This underestimation could potentially be attributed to unaccounted confounding variables or incomplete data, which lead to discrepancies between the predictions and observed values. Nevertheless, it is noteworthy that the 95% credible intervals of the average COVID-19 risk predictions encompass the observed average infection risks, suggesting that while there may be some deviation, the predictions are still within an acceptable range of uncertainty.

### Risk factor effects

Table 4 presents the results of our Bayesian regression analysis, showing the estimated regression coefficients and relative risks (RR) for each of the selected explanatory variables in relation to COVID-19 risk. The estimated relative risks and 95% credible intervals were computed by exponentially transforming the regression coefficients associated with the variables. Note that the relative risks relate to realistic increases in each variable, which are given in brackets in column 1 of the table.

**Table 4:** Estimated regression coefficients, relative infection risks and 95% credible intervals for the effects of each risk factor on COVID-19 infection risk. The relative risks relate to realistic increases in each covariate, which are given in brackets in column 1 of the table.

Variable	Regression coefficient	Relative risks (95% CI)
Annual household income ($\pounds$1000)	0.0008	1.0008 (1.0005, 1.0012)
Unemployment rate (1%)	0.0027	1.0027 (1.0024, 1.0030)
Log(population density)	0.0146	1.0146 (1.0129,1.0164)
Percent of Chinese (1%)	− 0.0160	0.9841 (0.9822, 0.9858)
Percent of Indian (1%)	− 0.0005	0.9995 (0.9992, 0.9998)
Percent of Pakistani (1%)	− 0.0016	0.9984 (0.9982, 0.9990)
Percent of Bangladeshis (1%)	− 0.0014	0.9986 (0.9982, 0.9990)
Percent of African (1%)	− 0.0093	0.9907 (0.9901, 0.9913)
Percent of Caribbean (1%)	0.0022	1.0022 (1.0009, 1.0036)
Percent of age 18–29 (1%)	− 0.0050	0.9950 (0.9947, 0.9954)
Percent of age 45–64 (1%)	0.0031	1.0031 (1.0024, 1.0039)
Percent of 65 years old and over (1%)	− 0.0066	0.9934 (0.9929, 0.9938)
Annual mean $\text {PM}_{2.5}$ (1 µg $\text {m}^{-3}$)	0.0125	1.0126 (1.0083, 1.0167)
Care home beds (0.01)	1.3295	1.0134 (1.0121, 1.0147)
Emergency facilities (TRUE)	0.0007	1.0007 (0.9958, 1.0057)

The table clearly demonstrates that the selected variables significantly contribute to the spatio-temporal variations of COVID-19 infection risk, with the exception of the emergency facilities variable because its 95% credible intervals include the null risk of 1. One of the main drivers of elevated infection risks is socioeconomic factor. We found that both annual household income and unemployment rate were associated with higher infection risk. Specifically, an increase of $\pounds$1000 in annual household income within an MSOA was associated with a 0.08% increased risk (RR = 1.0008, 95% CI 1.0005–1.0012), while an increase of 1% in unemployment rate was associated with a 0.27% increased infection rate (RR = 1.0027, 95% CI 1.0024–1.0030). Expectedly, the logarithm of the population density was positively correlated with the risk. A 1-unit increase in $\log (\text {population density})$ was found to be associated with a 1.5% rise in COVID-19 infection rate (RR = 1.0146, 95% CI 1.0129–1.0164). We also identified interesting patterns in the associations between ethnicity, age groups, and COVID-19 infections. Neighbourhoods with a greater percent of Chinese population tend to have lower risk of infections (RR = 0.9840, 95% CI 0.9823–0.9858). The Indian, Pakistani and Bangladeshi ethnic groups had a lower level of infections, with rates decreasing by 0.05%, 0.16% and 0.14%, respectively, for every 1% increase in the percentage of these groups within an MSOA. The African population was also associated with a decreased risk of COVID-19 infection (RR = 0.9907, 95% CI 0.9901–0.9913), whereas the Caribbean population had statistically significantly higher COVID-19 infection rates (RR = 1.0022, 95% CI 1.0009–1.0036).

Table 4 further reveals that infection risks tend to be lower in MSOAs with higher percentages of adults aged 65 years and older (RR = 0.9934, 95% CI 0.9929–0.9938), and those with higher percentages of adults aged 18–29 years (RR = 0.9950, 95% CI 0.9947–0.9954). Conversely, the population aged between 45 and 64 years old was positively correlated with infections, with a 1% increase being associated with a 0.31% higher rate. Additionally, there was a positive relationship between $\text {PM}_{2.5}$ concentrations and COVID-19 infections, with a 1 µg $\text {m}^{-3}$ increase in concentrations associated with between a 0.83% and a 1.67% increased rate (RR = 1.0126, 95% CI 1.0083–1.0167). Finally, increasing the number of care home beds per adult population (RR = 1.0134, 95% CI 1.0121–1.0147) was diagnosed positively linked to higher infection risks.

### Hotspots of COVID-19 infection rates

**Figure 3:**
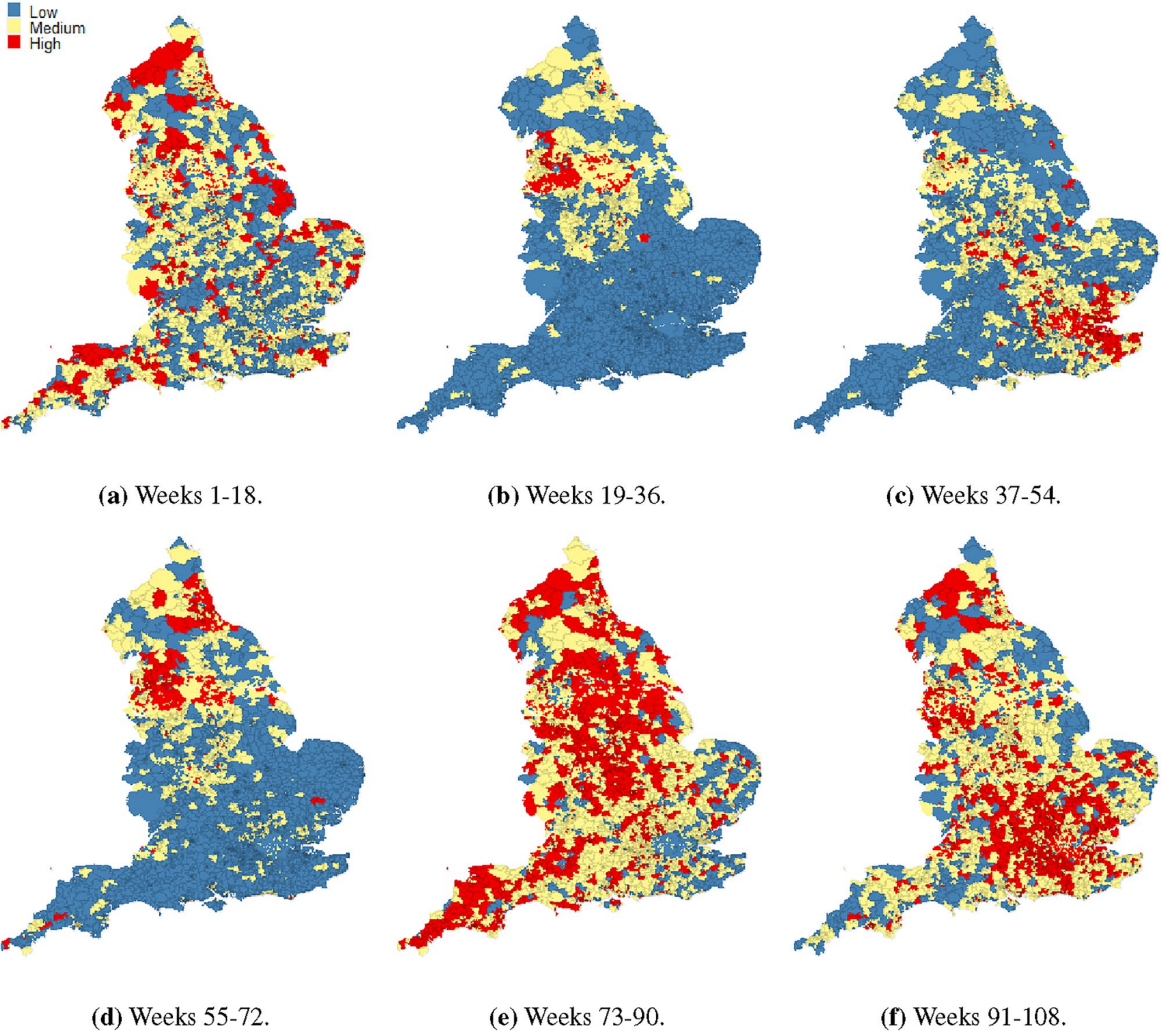
Maps showing the clusters that were formed according to the estimated infection risks at MSOA level during weeks 1–18 (7 March 2020–4 July 2020), weeks 19–36 (11 July 2020–7 Nov 2020), weeks 37–54 (14 Nov 2020–13 March 2021), weeks 55–72 (20 March 2021–17 July 2021), weeks 73–90 (24 July 2021–20 Nov 2021) and weeks 91–108 (27 Nov 2021–26 March 2022), respectively.

It is of interest to identify which MSOAs showed the high infection risks during the study period, and if there were any areas that consistently exhibited high risks. Here k-means clustering^60^ was used to identify the clusters of MSOAs with high and low infections. For ease of data visualization, we divided the 108-week time frame into six consecutive and non-overlapping intervals, each spanning 18 weeks. Since k-means clustering uses distance-based measurements to determine the similarity between data observations, we standardised the estimated infections risks during weeks 1 to 18, 19 to 36, 37 to 54, 55 to 72, 73 to 90 and 91 to 108, respectively, and then applied k-means clustering to the standardised risks. The MSOAs were assigned to between one and ten clusters based on their similarities in the estimated risks, and the optimal number of clusters was determined by using the elbow method^61^. Here the optimal number of clusters is 3 clusters for each time interval, and hence the MSOAs were classified into three distinct clusters that have a high, medium, and low level of infection risk, respectively. Figure 3 displays the spatial patterns of the cluster memberships of MSOAs for each time interval.

Figure 3 illustrates that the distribution of high infection risk was not consistent across different time periods and exhibited clustering patterns, indicating that certain areas were more significantly impacted by the pandemic than others. During weeks 1 to 18 (7 March 2020–4 July 2020), the highest infection rates were mainly concentrated in northern England, such as districts of Cumbria, Lancashire, Northumberland and Newcastle (County Durham, Gateshead, Sunderland), and in the far southwest, such as Devon. From week 19 to week 36 (11 July 2020–7 Nov 2020), hotspots for COVID-19 infection risks were highly clustered in a contiguous aggregation of MSOAs spanning from Lancashire, Manchester and Liverpool through to Bradford, Leeds, Kirklees and Sheffield. However, this trend moved to the southeast, particularly in London and some surrounding areas such as Kent and Essex in the following 18 weeks. During weeks 55 to 72 (20 March 2021–17 July 2021), the clusters of MSOAs with high infection rates were mainly in and around metropolitan areas for example Manchester, Liverpool, Newcastle, and North Tyneside. Interestingly, weeks 73 to 90 (24 July 2021–20 Nov 2021) showed a more dispersed distribution of hotspots, with hotspots scattered throughout England and no clear clustering patterns observed. However, there was a higher number of hotspots in central England and surrounds (Nottingham, Birmingham, Leicester, Stoke, Coventry, Leeds, Sheffield, Doncaster and Hull), as well as in the southwest (Bath, Bristol, Plymouth and Exeter). Finally, in the weeks 91 to 108 (27 Nov 2021–26 March 2022), high infection rates shifted towards the southeast of England, with the regions of Greater London, South East and East of England most affected. Despite the varying spatial patterns over time, the hotspots tended to cluster in urban and populous areas, particularly in the northwest, central, and southeast. The shifting patterns of infection risks demonstrate the dynamic nature of the pandemic, and the importance of monitoring temporal trends in different regions. Thus in the next section we investigated the temporal trends of COVID-19 infection risks in diverse regions of England.

### COVID-19 infection risk by region

**Figure 4:**
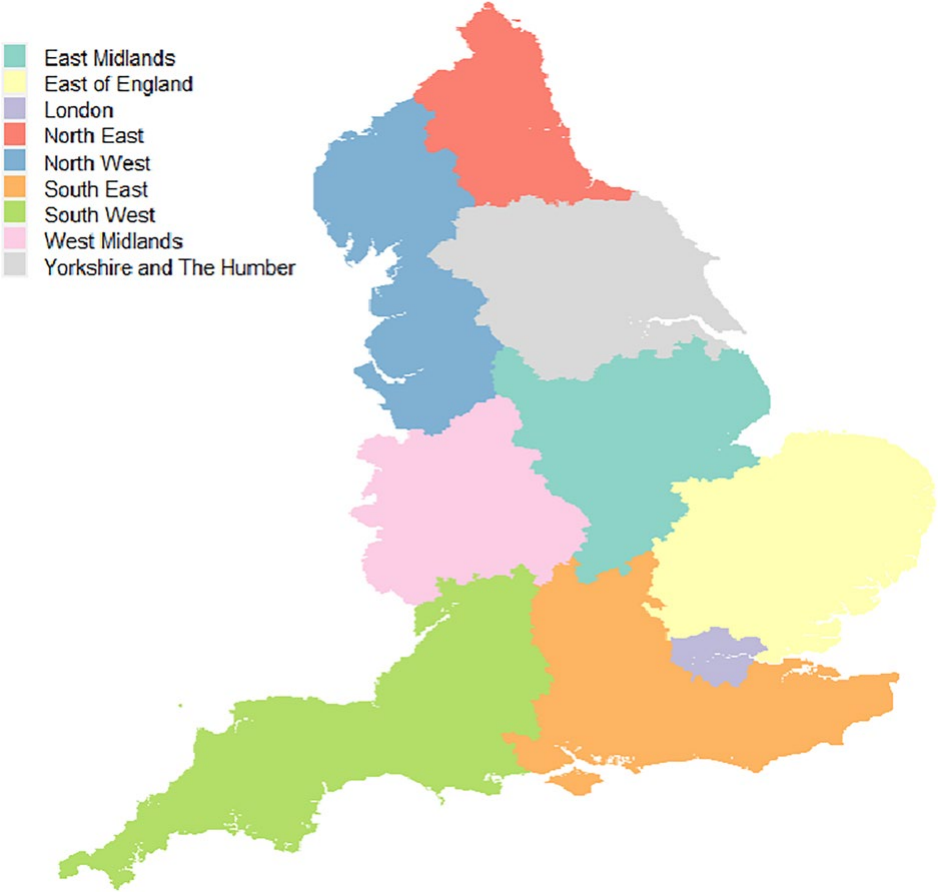
A map of the nine regions of mainland England.

**Figure 5:**
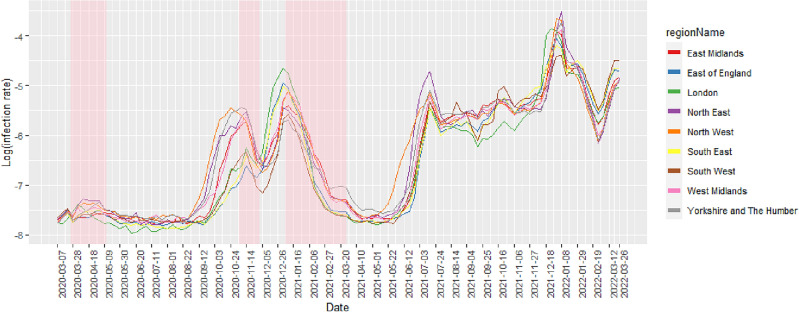
Population-based weighted averages of COVID-19 infection risks on the log scale by week and region in mainland England. Weeks of lockdown are highlighted in pink.

Differences in COVID-19 infection risk trends can be explored at a larger geographical scale by examining regional data, which provides a more aggregated view of the results. The 6789 MSOAs are nested exactly within nine regions of England, comprising North East, North West, Yorkshire and The Humber, East Midlands, West Midlands, East of England, London, South East and South West. Figure 4 provides a geographical representation of these nine regions. To characterise the temporal evolution of infection risk in each region, we used equation (6) to compute the risk of infection at the regional level. Figure 5 depicts the line plots of the population-based weighted averages of estimated infection risks after logarithm transformation by week and region. Here we chose to plot the log-infection risks instead of the infection risks themselves, as this provides a more suitable scale that allows for better visualisation of the temporal trend curves.

Figure 5 suggests that the national lockdown measures have been successful in containing the transmission of the virus, because in all regions the infection rates visibly declined following the initiation of any of the three lockdowns. During the first lockdown period, the Greater London region had the quickest decline in infection risk, while the risk in other regions, particularly East Midlands, took longer to reduce. After the lockdown was lifted, infection levels remained stable in most regions until August 2020, but increased notably from September 2020 onwards, with peaks observed in mid-November 2020 in all regions except for North West. When the second lockdown was in place, infection rates decreased in all regions, but remained higher in the northern (i.e., North East, North West) and central regions (i.e., Yorkshire and The Humber, East Midlands and West Midlands) of England. After the lockdown was lifted, the rates surged particularly in London, South East, and East of England due to the dominant Alpha variant, which was estimated to be more transmissible than preexisting variants^62^.

During the third lockdown, infection risks across England reduced substantially, with the Greater London region showing the most rapid decline as was the case in the first lockdown. A possible reason for this is that the percentage of key workers in London is relatively lower than that in other regions^63^. Key workers, such as healthcare workers and public transportation workers, are more likely to contract the virus as they continue to work and interact with the public during the imposition of restrictions. With fewer key workers in London, the lockdown measures may have been more effective and noticeable in reducing virus transmission compared to regions with larger numbers of key workers. There were no lockdown restrictions imposed from April 2021 to the end of the study, during which all regions experienced two infection peaks. One peak occurred in mid-July 2021, with rates ranging between 0.42% and 0.89%, while the other was in early Jan 2022, with rates ranging between 1.21% and 2.61%. Infection risks remained relatively stable between the peaks, with London having the lowest values. Infection levels sharply reduced from 8 Jan 2022, but rebounded by the end of Feb 2022. South West, South East, and East of England had the highest infection rates throughout the remainder of the study.

## Discussion

This study aimed to explore the spatio-temporal spread of COVID-19 infections in mainland England from 7 March 2020 to 26 March 2022, and its associations with socioeconomic, demographic and environmental factors. It can provide information for public health policies at the neighbourhood level, offering valuable insights for policymakers to optimise healthcare resources, establish targeted public health interventions, and improve epidemic prevention and control systems. Additionally, the study contributes new cases and knowledge to the growing body of research on the space-time transmission of COVID-19.

A Bayesian hierarchical spatio-temporal model was used to predict the COVID-19 infection risks based on weekly reported COVID-19 cases from 7 March 2020 to 26 March 2022 at MSOA level. Note that we utilised the COVID-19 data at MSOA level rather than the other geographical levels such as the Lower Layer Super Output Area (LSOA) level due to data availability. At the time of the analysis, the MSOA level was the smallest geographic unit for which both COVID-19 data and risk factors data were accessible from open sources. We did not downscale the MSOA geographical information to a higher-resolution geographical level, because the downscaling procedure will likely induce bias and inflate the reported credible intervals for the predicted infection risk^64^. Moreover, the MSOA level is commonly used in the literature for studying the small-areas COVID-19 transmissions^24^. To evaluate the model’s prediction performance, our Bayesian model was compared with the non-spatial regression model (OLS model) and the geographically weighted regression model (GWR model). The RMSE and MAE values of the Bayesian approach were lower than those of OLS and GWR models, indicating that the Bayesian model exhibited better prediction accuracy than the other models in this study.

The model estimation results showed that the level of spatial inequalities of COVID-19 infection risk increased over time, highlighting the need for effective strategies to address the disparities between different neighbourhoods. The COVID-19 infection risk in England exhibited spatio-temporal heterogeneity, with higher values observed during the months of July, December and January. These findings could be explained by the increased travel, outdoor activities, and social gatherings during the summer months, and holiday celebrations and family gatherings during the winter months. These factors could contribute to higher levels of interpersonal contact within a population, potentially leading to increased transmission risk. Besides, COVID-19 virus may be more transmissible in colder and drier conditions^8,65^, contributing to higher infection risks during the winter months. The infection risks were not evenly distributed across the country, with certain areas more vulnerable to the pandemic. Furthermore, the hotspots of infection risks exhibited clustered patterns that changed over time, with a higher frequency of occurrence in and around urban areas such as Newcastle, Manchester, Birmingham, Liverpool, Nottingham, Sheffield, Leeds, and London. The analysis of regional data indicated that although identical national lockdowns were announced across England, different regions displayed varying impacts in response to these measures. This variation could be influenced by factors such as the proportion of essential workers and the emergence of new variants of the virus. Moreover, it is important to note that different regions in England experienced slight variations in the start and end dates of these national lockdowns due to local epidemiological conditions and the tier system. The tier levels, ranging from Tier 1 (medium alert) to Tier 4 (very high alert), determine the extent of restrictions imposed on social interactions, businesses, and public spaces (https://www.ageuk.org.uk/information-advice/health-wellbeing/conditions-illnesses/coronavirus-guidance/local-lockdown-tiers/https://www.ageuk.org.uk/). Regions with higher infection rates and greater risk were typically assigned to higher tier levels, leading to more stringent measures and restrictions. Thus some regions may have entered lockdown earlier or experienced more prolonged periods of restrictions compared to those in lower tiers. Therefore, the observed differences in response to these measures may also be influenced by the region-specific timing and duration of the lockdown measures.

The analysis of socioeconomic, demographic, and environmental factors in relation to COVID-19 infections further indicated the key factors influencing the COVID-19 landscape in England. We found that MSOAs with higher annual household income had a higher infection risk, which is in agreement with previous investigations conducted in other countries or regions^21,66^. It is assumed that the influence of annual household income on the infections is related to the presence of a better network of health services, expanding the population’s access to carrying out diagnostic tests in the communities with high income, reducing underreporting, and contributing to increased COVID-19 incidence. Another socioeconomic indicator is unemployment rate, which was found to be positively associated with COVID-19 infection risk. Consistent findings have been found to exist in France and the United States^67,68^. A positive relationship was found between the infections and population density. We uncovered that the percent of Asians, including Chinese, Indian, Pakistani, and Bangladeshis, was negatively related to COVID-19 infection risk. This corroborates with the study conducted by Lee et al.^41^. Conversely, MSOAs with a higher proportion of Caribbean population had elevated risks. These relationships may be related to the ethnic differences in COVID-19 vaccine uptake^69^ and health behaviours (https://www.iser.essex.ac.uk/blog/2021/06/14/are-there-ethnic-differences-in-adherence-to-recommended-health-behaviours-related-to-covid-19https://www.iser.essex.ac.uk/blog/). The age composition of MSOAs was also found to impact COVID-19 infection levels. Higher proportions of adults aged 65 and older were related to lower infection rates. This association could be linked to factors such as higher vaccination rates among older adults, as they were among the first groups prioritised for COVID-19 vaccination in the UK. MSOAs with a higher percentage of young adults aged 18–29 years old showed a lower level of infection, while populations between 45 and 64 years old were positively associated with infection rates. Finally, elevated concentrations of $\text {PM}_{2.5}$ were found to be positively associated with COVID-19 infection risk, which is consistent with existing studies that have reported a positive relationship between increasing $\text {PM}_{2.5}$ concentrations and COVID-19 infections in various regions such as Ohio, Colorado, and Scotland^11,41,70^. This could be related to the increased susceptibility to respiratory infections such as COVID-19 given exposure to air pollution^71,72^. In addition, air pollution has been linked to underlying health conditions such as diabetes, cardiovascular, and respiratory diseases, which are known risk factors for severe COVID-19^73^. Therefore, it may be necessary to implement more rigorous COVID-19 prevention measures in areas with higher $\text {PM}_{2.5}$.

This study provides evidence that local rates of COVID-19 infections are influenced by patterns of household income, unemployment rate, population density, ethnic composition, age population structure and exposure to air pollution. It provides a scientific basis for accurately predicting COVID-19 response and targeting recovery efforts in England based on community-specific risk factors. It is important to consider these risk factors when developing effective control measures and allocating resources in different communities to control the pandemic spread. For example, public health interventions, such as promoting measures to reduce personal exposure to fine particle pollution could be implemented in specific neighborhoods at higher infection risk, providing better protection for vulnerable populations. Most importantly, we show that our BHM framework is an effective and powerful tool for modelling and understanding the risk factors that influence the infectious disease dynamics in general. Nevertheless, this study has some limitations. The most significant is its ecological design, where the unit of inference is the group of individuals living in each MSOA rather than having data for each individual. The aggregation of data at the geographic level can cause loss or concealment of certain details about individuals, resulting in ecological fallacy^74^ in the observed association. Thus, the estimated population-level associations should not be interpreted as cause-and-effect relationships at the individual level, because they may be influenced by factors such as within-area variation in either the exposure or the confounders. We used this ecological study design because the data required for an individual-level study were not available due to confidentiality reasons. We note, however, that this ecological approach has been predominant in the COVID-19 literature that focuses on exploring the distribution pattern of the pandemic and its influencing risk factors^2,75,76^. Our findings outlined above should be treated as indicative associations, rather than conclusive evidence of individual-level causation. To fully understand the causal relationships, further research, including tailored experimental designs combined with primary data collection, is necessary to establish causality definitively and investigate the mechanisms and biological pathways behind the observed associations. Secondly, since the annual household income data used were for 2018, the time gap between income data and other predictors and response data may influence the model estimation results. Thirdly, the reported COVID-19 infection cases were linked to the MSOA where the test was conducted, rather than the patient’s place of residence. This is a concern for metropolitan areas where a considerable number of patients could have been admitted or transferred to neighboring healthcare facilities before being tested. Fourthly, the infections data collected in the early phase of the pandemic were often underestimated due to a lack of testing, which could potentially lead to biased estimates and uncertainty in the model predictions. It is important to note that the number of reported COVID-19 cases is a function of the number of administered tests. Areas with higher testing rates may report more cases, leading to the perception of elevated infection rates, while areas with limited access to testing may underestimate their true case numbers. The uneven distribution of COVID-19 tests across regions and over time may affect the accuracy of infection risk estimation and cluster identification. We acknowledge that failing to consider this factor in our model presents a potential limitation to our study, which should be addressed in future research. Therefore, future work could incorporate testing information as a covariate in the model to control for potential biases arising from uneven testing over space and time, provided that such test data are available. The limitations of data quality and potential biases should be carefully considered when interpreting the modelling results for the initial pandemic stage.

We noted that in this study the effect sizes of the risk factors on the COVID-19 infection risk were relatively small. The small effect sizes are likely due to the small geographical level (MSOA) and the short time steps (weekly basis) for reporting the data. MSOAs represent small geographic areas with limited populations, therefore the spatial variations of risk factors and COVID-19 infections between English MSOAs naturally tend to be smaller than those between the larger geographical units such as counties or cities in other studies. Additionally, the use of weekly reporting interval tends to yield smaller risk variations compared to longer time steps. However, we believe that even these small effect sizes carry important information and implications for public health strategies. They contribute to our understanding of how specific risk factors influence infection risks at a fine-grained geographical level. By considering these effects, we can gain valuable insights for shaping localised interventions and preventive measures to reduce the spread of COVID-19 at neighbourhood level, and promote community health at this level of granularity. There are several avenues for future work. In this study we assume fixed effects of risk factors over time, however, we also acknowledge that the associations between these factors and the outcome variable may vary with time, thus there is potential to further enhance the model by incorporating spatially and temporally varying effects of risk factors. Meanwhile, since the spread of disease might be involved with varying spatial scales, future work would benefit from expanding our current model comparison to include the multivariate geographically weighted regression model. This model allows for analysing the relationships at different spatial scales^77^, providing a more comprehensive understanding of the complexities of the COVID-19 transmission. In addition, as stated previously, the associations found in the Bayesian model serve as indicative associations, rather than conclusive evidence of causation. There could be other factors that affect the identified relationships. Therefore, future applications could consider other potentially important predictors for COVID-19 infections such as comorbidities, population mobility and behavioural factors (e.g., alcohol consumption, smoking). The level of population immunity also plays a crucial role in shaping the disease dynamics and outcomes. To account for this factor in the model, vaccination data could be incorporated as a relevant variable, which is frequently cited as a proxy for population immunity. Lastly, extending the research to other countries and regions could offer comprehensive insights into the global spread of COVID-19 and inform the development of more targeted actions.

## Conclusions

This study demonstrates the effectiveness of a Bayesian hierarchical model that incorporates both risk factors and spatio-temporal random effects for understanding COVID-19 transmission. The results highlight the importance of considering socioeconomic, demographic, and environmental factors in analysing the spatio-temporal variations of COVID-19 spread in England. The framework provided here has the potential to serve as an early warning system for COVID-19 prevalence and assist policymakers in developing tailored public health interventions at a localised level. Furthermore, the proposed approach is also applicable to other similar research on spatio-temporal patterns of infectious diseases and their potential determinants.

## Data Availability

The data used in this study are available online from the UK COVID-19 dashboard (https://coronavirus.data.gov.uk/details/download), the 2021 UK census held by Office for National Statistics (https://www.nomisweb.co.uk/census/2021/bulk) and Department for the Environment, Food and Rural Affairs (https://uk-air.defra.gov.uk/data/pcm-data). **Accession codes** The main codes to complete this analysis are available from https://github.com/XueqingYin/COVID-19.git.
